# Effects of E-Liquids and Their Aerosols on Biofilm Formation and Growth of Oral Commensal Streptococcal Communities: Effect of Cinnamon and Menthol Flavors

**DOI:** 10.3390/dj12080232

**Published:** 2024-07-23

**Authors:** Nicole Christian, Daniel Burden, Alexander Emam, Alvin Brenk, Sarah Sperber, Michael Kalu, Giancarlo Cuadra, Dominic Palazzolo

**Affiliations:** 1 Biology Department, Muhlenberg College, Allentown, PA 18104, USA; nicolchr@umich.edu (N.C.); daniel.burden@cuanschutz.edu (D.B.); giancarlocuadra@muhlenberg.edu (G.C.); 2 School of Dentistry, University of Michigan, Ann Arbor, MI 48109, USA; 3 School of Dental Medicine, University of Colorado, Aurora, CO 80045, USA; 4 Debusk College of Osteopathic Medicine, Lincoln Memorial University, Harrogate, TN 37752, USA; alvin.brenk@yale.edu (A.B.); sarah.sperber@lmunet.edu (S.S.); michael.kalu@lmunet.edu (M.K.); 5 Yale New Haven Hospital, New Haven, CT 06510, USA

**Keywords:** commensal bacteria, oral cavity, electronic cigarettes, streptococci, E-liquids, electronic-cigarette-generated aerosol, bactericidal, hydrophobicity

## Abstract

(1) Background: The rise in electronic cigarette (E-cigarette) popularity, especially among adolescents, has prompted research to investigate potential effects on health. Although much research has been carried out on the effect on lung health, the first site exposed to vaping—the oral cavity—has received relatively little attention. The aims of this study were twofold: to examine the effects of E-liquids on the viability and hydrophobicity of oral commensal streptococci, and the effects of E-cigarette-generated aerosols on the biomass and viability of oral commensal streptococci. (2) Methods: Quantitative and confocal biofilm analysis, live–dead staining, and hydrophobicity assays were used to determine the effect on oral commensal streptococci after exposure to E-liquids and/or E-cigarette-generated aerosols. (3) Results: E-liquids and flavors have a bactericidal effect on multispecies oral commensal biofilms and increase the hydrophobicity of oral commensal streptococci. Flavorless and some flavored E-liquid aerosols have a bactericidal effect on oral commensal biofilms while having no effect on overall biomass. (4) Conclusions: These results indicate that E-liquids/E-cigarette-generated aerosols alter the chemical interactions and viability of oral commensal streptococci. Consequently, the use of E-cigarettes has the potential to alter the status of disease and health in the oral cavity and, by extension, affect systemic health.

## 1. Introduction

As the use of traditional tobacco-containing cigarettes has decreased, the use of electronic cigarettes (E-cigarettes) has become highly popular [1,2]. E-cigarettes were first introduced in China in 2003, where they gained attention as both a harm-reducing alternative to traditional smoking and as a smoking cessation tool [3,4]. Most E-cigarettes are composed of a battery, heating coil, E-liquid-filled cartridge, and mouthpiece [5]. The E-liquids typically consist of propylene glycol (PG), vegetable glycerin (VG), a plethora of flavoring agents [6], and variable concentrations of nicotine [7]. When the E-liquid is heated by the coil, it generates an aerosol that is inhaled by the user in the same manner as traditional cigarette smoke [5]. This is referred to as vaping.

Recently, there has been an alarming increase in the number of adolescents using E-cigarettes [8]. In fact, E-cigarettes have become the most commonly used tobacco product among adolescents [9]. In December 2018, the Surgeon General of the United States issued an advisory that declared the use of E-cigarettes among adolescents an epidemic [10]. The variety of available flavored E-liquids is one of the most commonly cited reasons among adolescents for use of E-cigarettes [11]. Another contributing factor to the rise in adolescent E-cigarette use is the robust advertisement targeting this group; for example, the packaging and flavoring are specifically designed to attract this group [2]. Many E-cigarette users have not previously used other tobacco products. This means that E-cigarette use is creating a new generation of nicotine dependence among teenagers and young adults who perceive vaping as less harmful than smoking traditional cigarettes [12].

Since E-liquids contain fewer substances and are non-combustible, E-cigarettes are marketed as a healthier alternative to traditional cigarettes or as an aid for smoking cessation [13,14]. However, evidence suggests that E-cigarettes may be just as dangerous as traditional cigarettes [15,16,17]. Vaping has been linked to adverse pulmonary and cardiovascular health conditions [18]. Some examples of short-term respiratory effects caused by E-cigarettes include transient lung inflammation [19] and increased airway resistance [20]. The flavoring components of E-liquids have been shown to cause the most detrimental effects [21,22]. Although there is much research regarding the effects of E-cigarettes on the lungs, there is relatively little research concerning their effect on the oral cavity.

The oral cavity has one of the highest concentrations of bacteria in the human body with over 700 species of microbes [23], second only to the microbiome in the gastrointestinal tract. The oral microbiome typically exists in the form of biofilms, which are communities of bacteria living adhered to oral surfaces, and plays a critical role in maintaining homeostasis and preventing disease in the oral cavity [24]. Many oral bacteria are commensals (symbiotic with their human host) [25]. Under healthy conditions, commensals compete with pathogenic bacteria and make it harder for the latter to survive in the biofilms. As a result, pathogens are not able to cause infection and disease unless they disturb the commensal interactions [26]. For example, a previous study from our group demonstrated that oral commensals, *Streptococcus gordonii* and *Streptococcus intermedius*, protect against the invasion of the periodontal pathogen *Porphyromonas gingivalis* into oral epithelial cells [27]. On the other hand, conditions such as smoking or the overuse of antibiotics negatively impact the composition and structure of the oral microbiome [28,29,30], which increases the risk of dysbiosis and oral disease [31]. Furthermore, the oral and systemic health are intimately linked: periodontal diseases have been connected to widespread systemic conditions, such as diabetes and cardiovascular diseases [31,32]. Therefore, any potential detrimental effects of E-liquids on the oral microbiome could result in consequences for oral and, by extension, systemic health.

Considering that the oral cavity is the first site of contact with E-cigarette-generated aerosols, the oral microbiota are particularly susceptible to the potential harm of E-liquids [33]. Previous studies from our group have compared the effects of cigarette smoke, flavorless and flavored E-liquids, and their aerosols on oral commensal bacteria: *S. gordonii*, *S. intermedius*, *Streptococcus oralis*, and *Streptococcus mitis* [34,35,36,37]. These species are early colonizers of the oral cavity and play an important role in establishing healthy oral biofilms and dental plaque [38,39,40]. Disturbances to these bacterial communities caused by E-liquids could interfere with the homeostasis of the oral cavity, which could prompt dysbiosis and lead to oral disease. Early studies from our group show that traditional cigarette smoke is vastly more detrimental to the growth of oral commensal streptococci compared to flavorless E-liquid or its aerosol [34,35]. Subsequent studies demonstrated that some E-liquids containing 25% flavor (*v*/*v*) have a significant dose-dependent inhibitory effect on the planktonic growth of oral commensal streptococci in batch cultures [36]. The most recent study from our group by Xu et al. (2022) [37] found that E-liquids with 25% flavors have significant inhibitory effects on biofilm formation along with the growth of single-species and multispecies communities of oral commensal streptococci. The flavors cinnamon and menthol were identified to have the most detrimental effects [37]. Additionally, the study by Xu et al. (2022) put forward the hypothesis that the effect of E-liquids is bactericidal, but additional experiments are required to substantiate this claim [37].

In the current article, we further analyze the effects of E-liquids containing either menthol or cinnamon on the four commensal streptococci aforementioned. As an extension of the Xu et al. (2022) study, we hypothesize that menthol- and cinnamon-containing E-liquids have a bactericidal effect and also alter the hydrophobicity of the bacterial surface [37]. In addition, this study also tests the effects of E-liquid aerosols on the formation and viability of multispecies biofilms. Understanding the mechanism of vaping-induced effects on oral bacteria is a critical step to understanding the potential impact on oral and systemic health.

## 2. Materials and Methods

### 2.1. Reagents and Supplies

All reagents and supplies for this study were obtained from ThermoFisher Scientific (Waltham, MA, USA), unless otherwise noted.

### 2.2. Bacterial Strains

The streptococcal strains tested were *S. gordonii* DL1, *S. mitis* UF2, *S. intermedius* 0809 and *S. oralis* SK139, which were generously donated by Dr. Robert Burne from the University of Florida, College of Dentistry in Gainesville, FL. All strains were grown in brain heart infusion (BHI) broth supplemented with 5 μg/mL of porcine hemin or on BHI agar. Growth conditions for all cultures in BHI broth or on BHI agar were 37 °C and 5% CO_2_, as previously described [27,34,35,36]. Bacterial stocks were stored at −80 °C; prior to experiments, their purity was confirmed through Gram staining and light microscopy.

### 2.3. Stock E-Liquids

**Figure 1:**
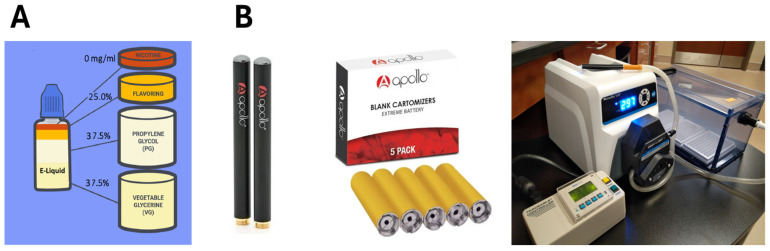
Description of E-liquid and apparatus. The E-liquid (**A**) and apparatus (**B**) used to expose streptococcal bacteria to E-cigarette-generated aerosol ± flavors are shown. In (**B**), the (left) shows the Apollo ECIG batteries; the (middle) shows the Apollo blank cartridges; the (right) shows the peristaltic pump, Traceable^TM^ controller, and exposure chamber. Note the Tygon S3^®^ tubing running from the E-cigarette to the pump and finally to the exposure chamber.

For E-liquid viability and hydrophobicity studies, the base flavorless solution of E-liquid was prepared using a 1:1 *v*/*v* ratio of PG and VG (Liquid Nicotine Wholesalers, Phoenix, AZ, USA) and then spiked with 20 mg/mL of (S)-(−)-nicotine, 99% (Alpha Aesar, Tewksbury, MA, USA). The stock flavors, which included cinnamon (Liquid Nicotine Wholesalers, Phoenix, AZ, USA) and menthol (Vapor Vapes, Sand City, CA, USA), were added to a final concentration of 25% (*v*/*v*) in the base flavorless E-liquid (Figure 1) following our previous protocols [36,37]. Stock E-liquid solutions for the E-cigarette-generated aerosol studies were prepared in the same manner except that no nicotine was added to the base E-liquid. After preparation, all E-liquids were stored at 4 °C.

### 2.4. Saliva Preparation

Saliva donations were collected from at least five healthy donors under IRB approval code Cuadra_S19_18. Following previous protocols [37,41], all participants met the following criteria: (i) non-smokers and non-vapers, (ii) healthy at the time of donation, (iii) had not taken antibiotics for at least 3 months prior to donation, and (iv) had not consumed any foods or drinks (aside from water) within two hours prior to donation. Raw saliva donations were stored at −20 °C before being thawed and mixed cold (ice bath). Dithiothreitol was added to a final concentration of 2.5 mM, and saliva was stirred on ice for approximately 10 to 15 min. The saliva was then centrifuged at 14,000× *g* for 45 min, and the supernatant was diluted 1:4 *v*/*v* with distilled water. The diluted saliva was then filter-sterilized through a 0.45 μm filter (Vacuum Filtration Systems, VWR). Sterile saliva was stored up to a year at −20 °C or up to two weeks at 4 °C before use.

### 2.5. Streptococcal Biofilm Exposure to E-Liquids

As previously described [37], 500 µL of processed saliva was added to each chamber of a 4-chamber slide and allowed to sit for 48 h at 4 °C to develop a salivary pellicle on the chambers’ surface. Frozen stocks of each of the four commensal streptococci were streaked on BHI agar and incubated at 37 °C and 5% CO_2_ overnight. Subsequently, they were inoculated in BHI broth and grown for an additional 24 h. After overnight incubation, batch cultures of each bacterial strain were diluted to an optical density (OD) of 1.0 at 595 nm wavelength. These samples were diluted 1:4 in BHI broth, and the four species of bacteria were mixed in a 1:1:1:1 ratio. Saliva was removed from the chamber slide, and the chambers were washed with 200 µL of phosphate-buffered solution (PBS). Two hundred microliters of mixed commensal streptococci were added to each chamber of the chamber slide and incubated at 37 °C and 5% CO_2_ for one hour to allow for adherence of bacteria to the salivary pellicle. After incubation, excess bacteria in each chamber were washed twice with 200 µL of PBS. Then, 50% BHI (diluted *v*/*v* with sterile water) was added to all chambers and incubated for 24 h at 37 °C and 5% CO_2_ to grow multispecies biofilms. The next day, all multispecies biofilms were washed twice with PBS. Then, 200 µL of 50% BHI (control); 50% BHI containing 5% flavorless E-liquid; or E-liquids ± 25% menthol or cinnamon flavors was added to the chambers and incubated for 3 h at 37 °C and 5% CO_2_.

### 2.6. Viability of Streptococcal Biofilm/Biomass after E-Liquid Exposure

Following the 3 h incubation, the multispecies streptococcal biofilms in the chamber slides were washed twice with 200 µL of PBS and stained with 5 μM of the LIVE/DEAD BacLight staining reagents from the bacterial viability kit (Cat No. L7012, Invitrogen, ThermoFisher) following the manufacturer’s instructions. The excess stain was washed twice with 200 µL of PBS. The biofilms were immediately imaged using a Carl Zeiss LSM880 laser scanning confocal microscope (Carl Zeiss Inc., White Plains, NY, USA) at 630× magnification with oil immersion. Excitation wavelengths of 485 nm and 535 nm were used, yielding green and red light emissions, respectively. Optical slicing was set to 1 μm, and Z-stacks were acquired at slow speed and high resolution. The three-dimensional images were taken and green/red pixel quantification was performed using ZEN 3.5 software (Carl Zeiss Inc., White Plains, NY, USA). The confocal microscope used in this study and the time for its use were generously provided by the Biological Sciences Department in the College of Arts and Sciences at Lehigh University (Bethlehem, PA, USA).

### 2.7. Hydrophobicity of Streptococcal Bacteria

All streptococci were grown in BHI agar and broth as indicated above. Working cultures were centrifuged and pellets were resuspended in PBS to an OD of 1.5 at 595 nm wavelength. Seven treatment groups were prepared: PBS control (later n-hexane control), PG, VG, stock menthol flavor, stock cinnamon flavor, menthol E-liquid (25% flavor), and cinnamon E-liquid (25% flavor). Each individual E-liquid component was systematically tested alongside the complete E-liquid to determine which component of the E-liquid was responsible for any significant change in hydrophobicity. Seven 600 μL aliquots (one for each treatment group) for each of the four bacterial strains set to OD 1.5 were centrifuged at 15,000× *g* for 10 min. The supernatants were discarded, and the pellets were resuspended in 100 μL of each treatment followed by incubation for 15 min at 37 °C. After incubation, 500 μL of PBS was added to each bacteria/treatment combination and centrifuged at 15,000× *g* for 10 min. The supernatant was discarded, and all pellets were resuspended in 600 μL of PBS (aqueous phase) and 600 μL of n-hexane (organic phase) followed by 30 s of vortexing. The purpose of this is to allow hydrophobic bacteria to move into the organic phase and hydrophilic bacteria to end up in the aqueous phase. Subsequently, 500 μL of the aqueous layer (hydrophilic bacteria in PBS) for each bacteria/treatment sample was pulled out and diluted 1:2 in PBS. The OD of these samples was measured. Since the stock cinnamon flavor and cinnamon E-liquid treatments had an inherent amber background, blanks were made following the steps listed above using the treatments without bacteria. Since the stock menthol flavor and menthol E-liquid treatments crystallize at room temperature [42], blanks were also made for these conditions using the same procedure listed above without bacteria. The final absorbance readings for the cinnamon flavor, cinnamon E-liquid, menthol flavor, and menthol E-liquid treatments were calculated by subtracting the absorbance of the blank from the original readings. The reciprocal absorbance was utilized such that a higher absorbance value indicates an increase in hydrophobicity. The (n-hexane) control was utilized as a baseline hydrophobicity for each bacterial species.

### 2.8. Streptococcal Biofilm/Biomass Exposure to E-Cigarette-Generated Aerosols

A Masterflex model 77200-60 L/S peristaltic pump (Vernon Hills, IL, USA) was used to transport generated aerosol through 100 cm of Masterflex Tygon S3^®^ B-44-4X (ThermoFisher Scientific, Waltham, MA, USA) precision tubing (ID = 8.0 mm, 1.6 mm wall thickness) to an exposure chamber (Kent Scientific Corporation, Torrington, CA, USA). The dimensions of the exposure chamber are 12” L × 6” W × 6” H. The chamber is constructed of acrylic with a slider top to allow access to biofilm samples and an inlet and outlet for aerosol flow. One end of the Tygon S3^®^ tubing is connected to a “cigalike” E-cigarette (Apollo Future Technology, Inc, Las Vegas, NV, USA), and the other end of the tubing is connected to the inlet of the chamber. The peristaltic pump is positioned inline approximately 25 cm from the E-cigarette. The Apollo E-cigarette [43] consists of a battery (≈4.2 V/250 mAh), which lasts for ≈200 puffs when fully charged, and a blank cartridge (with resistance coil ≈ 2.0 Ω and power output of ≈ 8.8 Watts). The E-cigarette is self-actuated by negative pressure created whenever the pump is turned on. The cartridge is filled with in-house E-liquid consisting of PG and VG (1:1 *v*/*v*) ± 25% stock flavoring agents (menthol or cinnamon). Puff topography follows Cooperation Centre for Scientific Research Relative to Tobacco (CORESTA) guidelines of Method No. 81 (i.e., two 3 s puffs per minute with flow rate of 55 mL/puff) [44]. Before each experiment, the pump flow rate was equilibrated to 1.1 L/min (equivalent to 55 mL/puff) using an Aalborg GFM flow meter (Orangeburg, NY, USA), and the puff time was programed using a Fisherbrand^TM^ Traceable controller (ThermoFisher Scientific, Waltham, MA, USA). All experiments were conducted within a P20 Purair ductless fume hood (Airscience, Fort Meyers, FL, USA) with a high-efficiency particulate (HEPA) filter. Figure 1 illustrates the E-liquid and apparatus used in all puffing experiments.

### 2.9. Crystal Violet Quantification of Streptococcal Biofilm Biomass after Aerosol Exposure

Sterile plastic coverslips (13 mm diameter) were bathed in 1 mL of sterile human saliva for 48 h at 4 °C in sterile 12-well plates (1 coverslip/well). Starter overnight cultures of all bacterial strains were standardized to an OD of 1.0 at a 595 nm wavelength and diluted 1:4 with BHI broth. After standardization, 100 μL of each diluted culture was seeded separately on to the saliva-coated sterile plastic coverslips and placed in a fresh sterile 12-well plate (one coverslip/well). Additionally, 100 μL of a 1:1:1:1 mixture of all four standardized and 1:4 diluted streptococcal species was also seeded on saliva-coated sterile plastic coverslips and placed into a fresh sterile 12-well plate. Bacteria, individually or as a multispecies, were allowed to adhere to the surface of the coverslips for 1 h at 37 °C, 5% CO_2_, and the excess unbound bacteria were washed three times with 1 mL of sterile PBS. The 12-well plates containing the coverslips were then exposed to air (control) or ECIG-generated aerosol ± flavors. Exposure treatments consisted of 0, 15, 30, and 45 puffs of aerosol. Following aerosol exposure, 1 mL of 50% BHI broth (*v*/*v* in sterile water) was added to each well of the 12-well plate, ensuring that exposed coverslips were completely submerged. The 12-well plates were subsequently incubated for 24 h at 37 °C, 5% CO_2_ to allow for biofilm growth on the coverslips. At the end of the 24 h incubation period, the coverslips were washed three times with 1 mL PBS to remove excess unbound bacteria and placed into a new 12-well plate. Five hundred microliters of a 5% crystal violet solution were then added to each well and allowed to stain the bacterial peptidoglycan for 5 min. The coverslips were then rinsed up to seven times with 0.5 mL of deionized water until the only crystal violet remaining was within the bacterial cell wall. The coverslips were placed in a fresh 12-well plate. Next, 500 µL of 95% ethyl alcohol was added to all the wells followed by gentle shaking for 30 s to allow for complete extraction of the crystal violet from within the bacteria. The liquid in the wells was pipetted up and down a few times to ensure all the stain came out of the bacteria. From each well, 150 µL of the ethyl alcohol/crystal violet mixture was transferred to a 96-well plate. In addition, 150 µL of 95% ethyl alcohol was added to four more wells of the 96-well plate and served as zeroing blanks. The 96-well plate was placed into a Gen 5 plate reader (BioTek, Winooski, VT, USA) and absorbance measured at 595 nm wavelength. Absorbance readings served as an index of biofilm biomass.

### 2.10. Fluorescent Microscopy of Streptococcal Biofilm/Biomass after Aerosol Exposure

Bacterial biofilms (individual or as a multispecies combination) on coverslips were prepared and exposed to aerosol (0 and 45 puffs of air, or aerosol ± flavors only) as described above. However, following the 24 h incubation period, coverslips were washed three times with 1 mL PBS, then placed into a new 12-well plate. They were fixed with 4% formaldehyde for subsequent DNA staining with 5 µM SYTO 59 Red in PBS for 30 min at room temperature. After staining with SYTO 59, the coverslips were removed from the 12-well plates and mounted on glass slides with Permount^®^ for later fluorescent imaging. Each coverslip was photographed at 1000× total magnification (1920 × 1080 resolution) using a Nikon Eclipse TE2000-U microscope (Nikon, Melville, NY, USA) with an X-Cite 120 fluorescence illumination system (EXFO, Québec City, QC, Canada). The percent of bacterial biofilm biomass per image was determined using the public domain software ImageJ 1.53t (National Institutes of Health, Stapleton, NY, USA) in which color images (Red, Blue, and Green = RBG) were first converted to 8-bit black and white (B&W) images and then the percent of white pixels in each image was calculated. The percentage of white pixels represented the biofilm biomass.

### 2.11. Crystal Violet Quantification of Multispecies Streptococcal Biofilm Biomass Exposed to Aerosol before and after 24 h Incubation

One hundred microliters of saliva were placed in five wells of the first row of 12 sterile 96-well plates for 48 h at 4 °C. Starter overnight cultures of all bacterial strains were standardized to OD = 1.0 at 595 nm wavelength and diluted 1:4 with BHI. After standardization, 100 μL of a combination of all four streptococcal species (1:1:1:1) was seeded in the saliva-coated wells of the 96-well plates. The multispecies streptococcal bacteria were allowed to adhere to the bottom of the wells for 1 h at 37 °C, 5% CO_2_, and the excess unbound bacteria was washed three times with 200 µL of sterile PBS. At this point, the multispecies biofilms were exposed to 45 puffs of air, or E-cigarette-generated aerosol ± flavors. They were then incubated for 24 h (pre-24 h incubation group) or allowed to incubate for 24 h and then exposed to 45 puffs of air, or E-cigarette-generated aerosol ± flavors (post-24 h incubation group). Two additional groups, 0-puff and 0-puff with 5% peroxide, served as controls. During exposures to air or aerosols, biofilms were always exposed without 50% BHI. During 24 h incubation, biofilms were always covered with 100 µL of 50% BHI. Following exposure/incubation treatment, the multispecies bacteria were washed three times with 200 µL of PBS and then stained with 100 µL crystal violet solution for 5 min. The wells were then rinsed up to seven times with 200 µL of deionized water until the only crystal violet remaining was within the bacteria. Next, 200 µL of 95% ethyl alcohol was added to all the wells followed by gently shaking the plates for 30 s to allow for complete extraction of the crystal violet from within the bacteria. The liquid in the wells was pipetted up and down a few times to ensure all the stain came out of the bacteria. From each well, 150 µL of the ethyl alcohol/crystal violet mixture was transferred to a single fresh 96-well plate. In addition, 150 µL of 95% ethyl alcohol was added to 10 more wells of the 96-well plate and served as zeroing blanks. The 96-well plate was placed into a Gen 5 plate reader (BioTek, Winooski, VT, USA) and absorbance measured at a 595 nm wavelength. Absorbance readings served as an index of biomass.

### 2.12. Viability of Multispecies Streptococcal Biofilms Exposed to Aerosol before and after 24 h Incubation

One hundred microliters of saliva were placed in 10 wells (B2–B11) of six sterile, black, clear, flat-bottomed 96-well plates for 48 h at 4 °C. Starter overnight cultures of all bacterial strains were standardized to an OD = 1.0 at 595 nm wavelength and diluted 1:4 with BHI. After standardization, 100 μL of a combination of all four streptococcal species (1:1:1:1) was seeded in the saliva-coated wells. The multispecies bacteria were allowed to adhere to the bottom of these wells for 1 h at 37 °C, 5% CO_2_. Following incubation, the excess unbound bacteria in each well were washed 3 times with 200 µL of sterile PBS. At this point, the multispecies communities were exposed under two conditions: (1) 45 puffs of air or E-cigarette-generated aerosol ± flavors followed by incubation for 24 h (pre-24 h incubation group), or (2) 24 h incubation followed by 45 puffs of air or E-cigarette-generated aerosol ± flavors (post-24 h incubation group). Two additional groups, 0-puff and 0-puff with 5% peroxide, served as controls. During exposures to air or aerosols, biofilms were always exposed without 50% BHI. During 24 h incubation, biofilms were always covered with 100 µL of 50% BHI. Following exposure/incubation, the multispecies bacteria in all wells were stained according to instructions of the Invitrogen™ LIVE/DEAD™ BacLight™ Bacterial Viability Kits, Cat No. L13152 (ThermoFisher Scientific, Waltham, MA, USA). The standard curves for these assays were generated from individually standardized streptococcal bacteria (OD = 0.8 to 1.0). After standardization, each strain of bacteria was centrifuged at 12,000× *g* for 8 min and the pellets resuspended in 1 mL of PBS. This was repeated two more times. After the third centrifugation, the resuspended bacterial pellets were combined in 1:1:1:1 ratio. The combined bacteria were then standardized to OD = 1.0. Fifty percent of the combination streptococcal mixture was placed on ice, while the remaining 50% of the streptococcal mixture was heat killed (30 min at 70 °C) using a Fisher Scientific heat/cool thermal mixer (ThermoFisher Scientific, Waltham, MA, USA). The standards consisted of combining 100, 90, 50, 10, and 0% live bacteria with 0, 10, 50, 90, and 100% dead bacteria, respectively. The standard curve was derived from the ratio of fluorescent live bacteria to fluorescent dead bacteria. Fluorescence for live bacteria was determined at an excitation/emission of 485/530 nm, whereas fluorescence for dead bacteria was determined at an excitation/emission of 485/630 nm using a Synergy H1 (Bioteck, Winooski, VT, USA) microplate reader. One hundred microliters of each standard were placed in a sterile, black, clear, flat-bottomed 96-well plate (*n* = 4). Standards and samples were stained with 100 µL of the provided live/dead stains (final volume = 200 µL for each well) and allowed to incubate in the dark at room temperature for at least 15 min prior to reading.

### 2.13. Statistical Analysis

All data were expressed as means ± standard error of the means (SEM), and all comparisons were made using either one-way ANOVA or two-way ANOVA followed by a Bonferroni multiple comparison test where significance was achieved when *p* < 0.05. Version 5 of Prism (GraphPad Software, San Diego, CA, USA) was used to perform all statistical calculations and to generate all graphs.

## 3. Results

### 3.1. Biofilm Biomass Viability after E-Liquid Exposure

**Figure 2:**
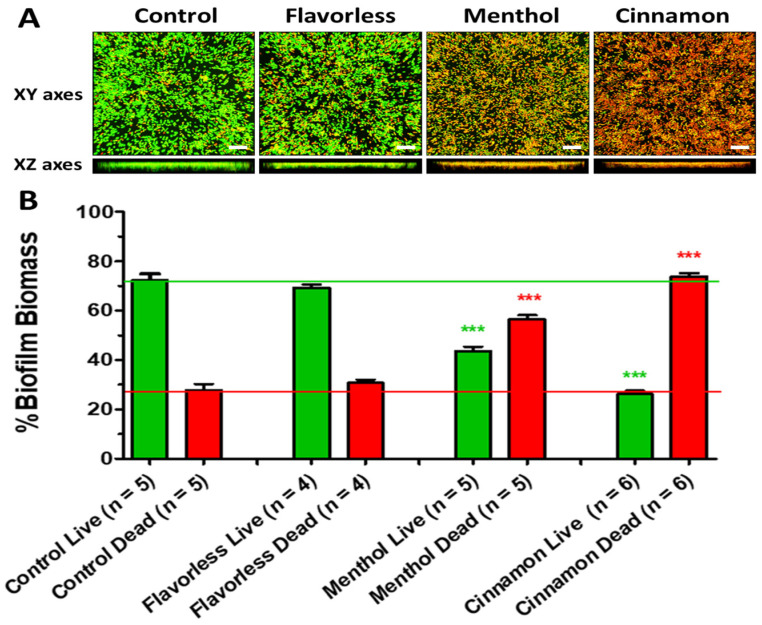
E-liquid treatments and live/dead stain. Viability of biofilm biomass exposed to BHI media alone (control) or BHI and 5% E-liquids ± 25% flavors. The medium is a 1:1 *v*/*v* mixture of BHI and distilled water. Representative confocal micrographs at 630× total magnification (XY); the white horizontal bars on the xy graphs indicate 20 µm. The Z-axis measures 8 to 10 µm in biofilm height (**A**). Quantification of percent live/dead biofilm biomass (**B**). Each bar represents the mean ± SEM (*n* = 4 to 6). Significance was determined using one-way ANOVA followed by a Bonferroni multiple comparison test. Green *** = *p* < 0.001 from live control and red *** = *p* < 0.001 from dead control.

In order to assess the impact of menthol- and cinnamon-containing E-liquids on the viability of multispecies biofilms formed on salivary pellicles, the biofilms were exposed to E-liquids with and without 25% menthol or cinnamon. In Figure 2A, confocal images depict viability for the multispecies biofilms (green = live; red = dead) treated with E-liquids ± flavors, while in Figure 2B, these images are quantitated using ImageJ. Confocal images of biofilms treated with the control and flavorless conditions indicate that the base components of E-liquids (nicotine, PG, and VG) exert little to no bactericidal effect on the oral commensal streptococci tested since the imaged biofilms reveal mostly live cells and fewer dead cells. In contrast, quantitation of the red/green cells in the biofilms exposed to both menthol- or cinnamon-flavored E-liquids significantly (*p* < 0.001) enhanced bacterial killing from 27.7% in the control group to 56.3% in the menthol-treated group and 74.8% in the cinnamon-treated group (Figure 2). These results indicate that menthol and cinnamon flavors have a bactericidal effect on these multispecies communities of oral commensal streptococci.

### 3.2. Hydrophobicity Assay

**Figure 3:**
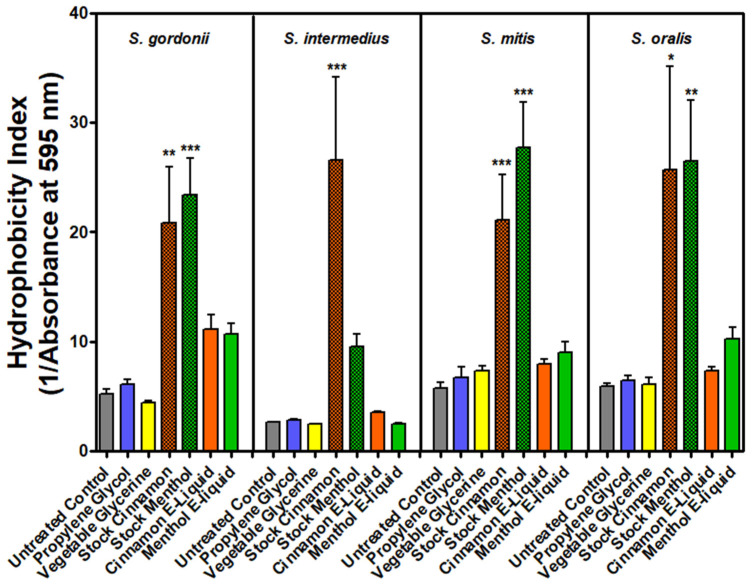
E-liquid treatments and hydrophobicity assay. Hydrophobicity, tested with n-hexane, after exposure of oral commensal streptococci to PG, VG, stock menthol, stock cinnamon flavors, E-liquid + 25% cinnamon, and E-liquid + 25% menthol. The control indicates baseline hydrophobicity for each of the bacterial species. Hydrophobicity is indexed by the reciprocal value of absorbance (595 nm) of the aqueous fraction, where an increase of 1/absorbance equates to an increase in hydrophobicity. Each bar represents the mean ± SEM (*n* = 6). Significance from the control was determined using one-way ANOVA followed by a Bonferroni multiple comparison test. * = *p* < 0.05, ** = *p* < 0.01, and *** = *p* < 0.001.

Hydrophobicity is an important property in biofilm formation. Weak chemical receptor/ligand interactions (electrostatic, van der Walls, hydrogen bonding, and hydrophobic interactions) are essential for bacteria–bacteria and host–bacteria crosstalk. In general, any alterations in hydrophobicity may lead to changes on bacterial surfaces, which could affect biofilm formation, interactions with the host, or microbial survival. In order to determine if E-liquid-treated oral commensal bacteria are more or less hydrophobic, bacteria were exposed to individual E-liquid components or complete E-liquids and allowed to partition between aqueous and organic solvents. Results were examined by measuring the OD of each condition within the aqueous fraction. As depicted in Figure 3, the baseline hydrophobicities of the four strains of oral commensal streptococci tested were established using the organic solvent, n-hexane, as a control. Control results indicated that there was no significant difference in hydrophobicity between the four oral commensal streptococci. In contrast, treatment with the stock menthol or cinnamon flavors resulted in increases in hydrophobicity across all four species. After cinnamon treatment, the increase observed, in comparison to controls, was statistically significant (*p* < 0.05) for *S. intermedius*, *S. gordonii*, and *S. oralis*. Menthol treatment significantly (*p* < 0.01) increased hydrophobicity on *S. gordonii* and *S. mitis*. Exposure to PG and VG resulted in no change in hydrophobicity as compared to the control across all four species. For the menthol and cinnamon E-liquid treatments, some species displayed a slight increase in hydrophobicity; however, this change was not statistically significant. Overall, these results indicate that the stock cinnamon and menthol flavors increase the hydrophobicity for the oral streptococci, while the E-liquids with these flavors (25%) result in a slight increase (not significant) in hydrophobicity. PG and VG have no effect.

### 3.3. Crystal Violet Quantification after Aerosol Exposure

**Figure 4:**
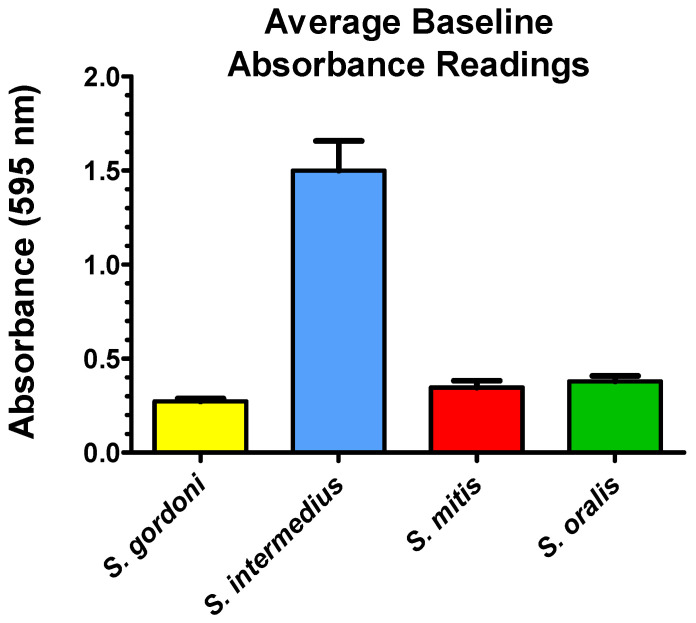
Baseline single-species biofilm biomass. Average baseline (control) of crystal violet absorbance for single-species biofilms. Each bar represents the mean ± SEM (3 replicate experiments, *n* = 4 per replicate, total = 12).

In order to establish the baseline of biofilm biomass, all species were grown as single-species biofilms, and their crystal violet staining levels were determined by absorbance (595 nm). Figure 4 depicts average baseline crystal violet absorbance readings for all four streptococcal species; these values represent controls. *S. intermedius* retained more crystal violet than the other three streptococcal species. From this point on, all absorbance results of crystal violet quantification will be expressed as a percent change from control.

**Figure 5:**
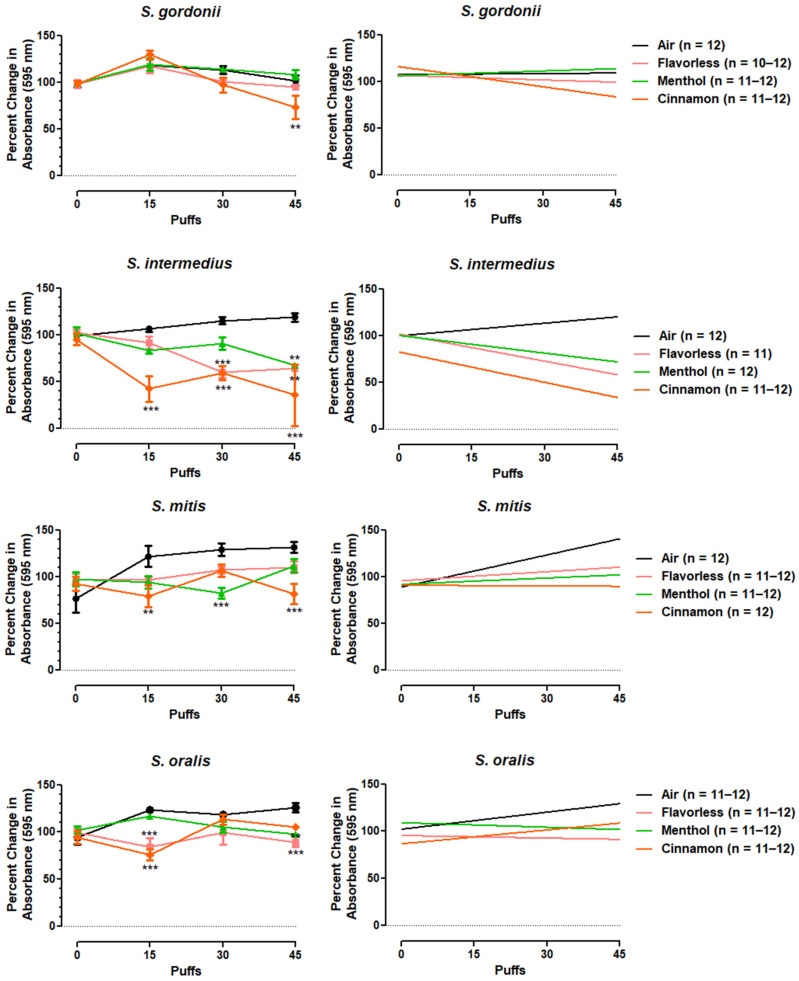
Aerosol-treated single-species biofilm biomass. Left-side graphs give a quantification of the oral commensal streptococcal biofilm biomass, as indexed by absorbance (595 nm) of crystal violet, after exposure to air or E-cigarette-generated aerosol ± flavors (menthol or cinnamon). Each point is the mean ± SEM (*n* = 11 to 12). Statistical significance was determined by two-way ANOVA with Bonferroni post hoc analysis comparing exposures of air to E-liquid ± flavors after 0, 15, 30, or 45 puffs. ** = *p* < 0.01, *** = *p* < 0.001. The right-side graphs display trend lines (slope) of the same data displayed in the left-side graphs.

In order to measure the effects of air and E-cigarette-generated aerosols ± flavors (menthol or cinnamon) on single- and multispecies biofilms of oral commensal bacteria, crystal violet staining was used to quantitate biofilm biomass as a function of treatment with aerosolized E-liquid ± flavors. Figure 5 shows the effects of air and E-cigarette-generated aerosol ± flavors (menthol or cinnamon) after 0, 15, 30, and 45 puffs on biofilm formation for all four streptococcal species along with their corresponding linear regressions. Table 1 shows the linear regression statistics. Compared to the air control, flavored E-cigarette-generated aerosols decreased the biofilm/biomass of all bacterial species, except for *S. gordonii* exposed to menthol. Flavorless E-cigarette-generated aerosol also decreased the biofilm/biomass of *S. intermedius* and *S. oralis*. While these decreases were not puff-dependent, linear regression analyses revealed significant decreases in biofilm biomass for all four species (Table 1). *S. gordonii* exposed to cinnamon aerosol shows a significantly (*p* < 0.01) lower negative slope as compared to the air control. Similarly, the regression lines for *S. oralis* exposed to flavorless and menthol aerosols show significantly (*p* < 0.01) lower negative slopes as compared to the air control. The negative slopes of the regression lines for *S. intermedius* and *S. mitis* exposed to flavorless, menthol, and cinnamon aerosols are all significantly (*p* < 0.01) lower as compared to the air control. In general, E-cigarette-generated aerosol ± flavors tend to decrease biofilm biomass for all bacteria evaluated. This was especially true for *S. intermedius*, where the R^2^ values for the air, flavorless, menthol, and cinnamon regression lines were 0.98, 0.83, 0.72, and 0.62, respectively (Table 1).

**Figure 6:**
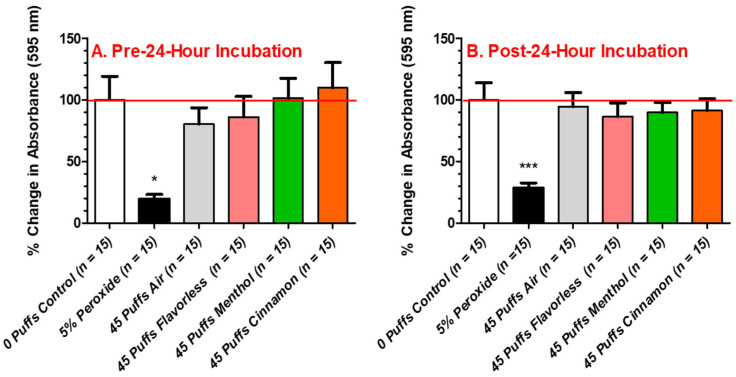
Aerosol-treated multispecies biofilm biomass. Quantitation of multispecies streptococcal biofilm biomass exposed to 45 puffs of air or E-cigarette-generated aerosol ± flavors (menthol or cinnamon), as indexed by absorbance of crystal violet before (**A**) and after (**B**) 24 h incubation. Each bar represents the mean ± SEM (3 replicate experiments, *n* = 5 per replicate, total *n* = 15). Statistical significance was determined by one-way ANOVA with Bonferroni post hoc analysis, where * = *p* < 0.05 and *** = *p* < 0.001 as compared to 0-puff control. The red line represents the average baseline absorbance reading for the 0-puff control.

To evaluate the effects of aerosols ± menthol and cinnamon on multispecies biofilm formation, bacteria were seeded on saliva-coated surfaces and immediately exposed to 45 puffs of aerosol ± flavors before growing (pre-24 h incubation). In contrast, to evaluate the effects of aerosols ± flavors after multispecies biofilm growth (post-24 h incubation), biofilms were exposed to 45 puffs of aerosol ± flavors. Figure 6 depicts the effects of 45 puffs of air and E-cigarette-generated aerosol ± flavors (menthol or cinnamon) on biofilm biomass formation (crystal violet absorbance readings expressed as percent change from 0 puffs) of the multispecies community of all four streptococcal bacteria. Exposure to air and the E-cigarette-generated aerosols ± flavors failed to hinder biofilm formation in the multispecies community, regardless of whether exposure preceded (Figure 6A) or followed 24 h incubation (Figure 6B). As expected, peroxide, as a positive control for inhibition of bacterial growth, significantly inhibits multispecies biofilm biomass formation. These results indicate that any decrease in biofilm biomass noted in Figure 5 by the individual streptococcal species is lost when these bacteria are grown as a multispecies community. Furthermore, the timing of exposure (i.e., pre- or post-24 h incubation) had no effect on the overall biomass of these biofilms.

### 3.4. Fluorescent Microscopy Analysis after Aerosol Exposure

**Figure 7:**
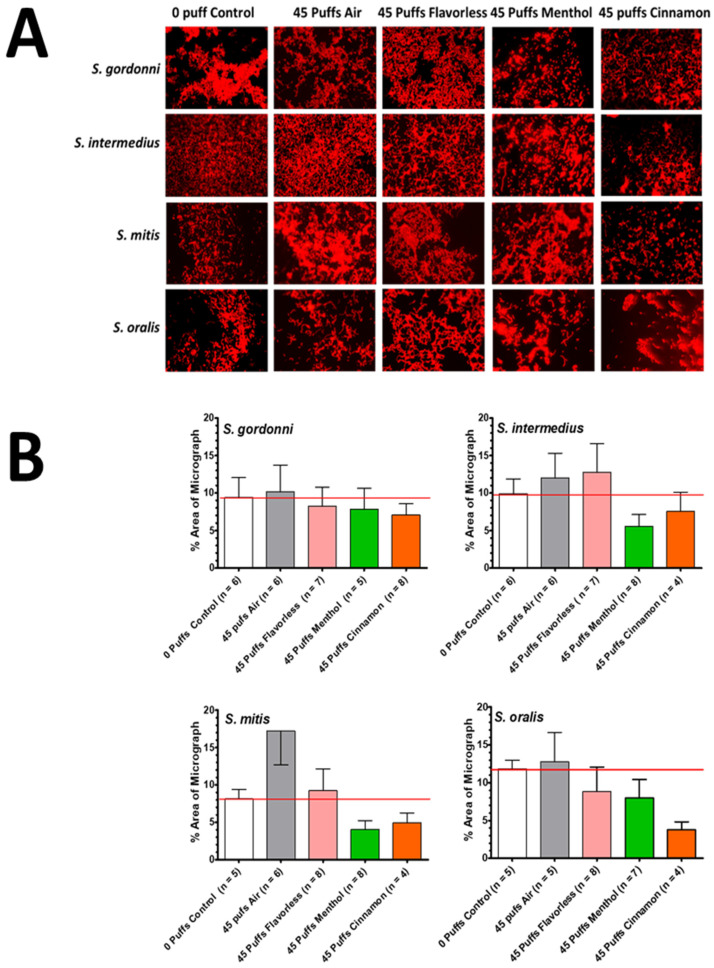
Aerosol-treated single-species biofilm microscopy and quantitation. Depiction of oral commensal single-species streptococcal biofilms using the DNA stain, SYTO 59 Red. (**A**) Representative biofilms are shown comparing 0-puff control for *S. gordonii*, *S. intermedius*, *S. mitis,* and *S. oralis* with 45 puffs of air, or E-cigarette-generated aerosol ± flavors. Each micrograph was photographed at 1000× total magnification (1920 × 1080 resolution) using a fluorescent microscope and converted to 8-bit black and white images (not shown). (**B**) The percentage of the biofilm biomass was calculated on the black and white images using ImageJ and is shown as % area of micrograph. Each bar represents the mean ± SEM (at least four random micrographs). The red line represents the average biofilm percentage of the 0-puff control.

Fluorescence microscopy was used to further investigate the effects of air and E-cigarette-generated aerosol ± flavors (menthol or cinnamon) by characterizing the biofilm architecture of single-species biofilms after 45 puffs of aerosol exposure. Representative images of each individual streptococcal biofilm (on coverslips) after exposure to 0 puffs (control) or 45 puffs of air or E-cigarette-generated aerosol ± flavors, stained with SYTO 59 Red, are shown in Figure 7A. No discernable differences in biofilm architecture for each of the streptococcal species is visually apparent between 0 and 45 puffs of air or E-cigarette-generated aerosols ± flavors. Furthermore, the biofilms vary considerably within each treatment group for each bacterial species. A random sampling of four to eight coverslips for each bacterial species exposed to the various treatment groups was used to quantitate the percent area of biofilms in the micrographs using ImageJ, as shown in Figure 7B. No significant differences in percent area of micrographs between 0 puffs and 45 puffs of air or the E-cigarette-generated aerosols ± flavors are indicated. Note that black and white converted images for ImageJ quantitation are not shown in Figure 7.

**Figure 8:**
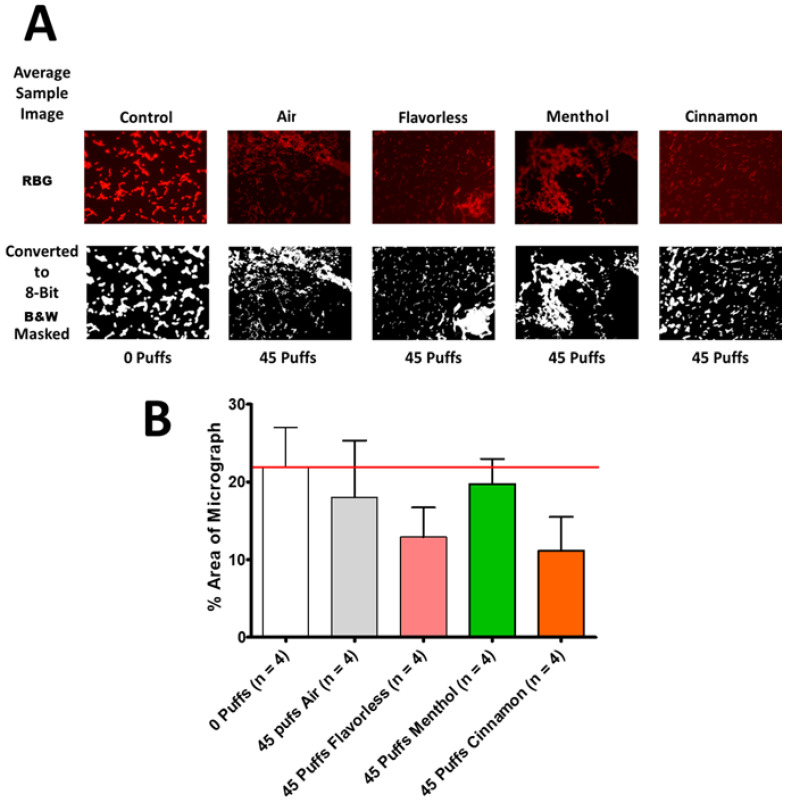
Aerosol-treated multispecies biofilm microscopy and quantitation. Depiction of oral multispecies (*S. gordonii*, *S. intermedius*, *S. mitis,* and *S. oralis*) streptococcal biofilms using the DNA stain, SYTO 59 Red. (**A**) Representative biofilms are shown comparing 0-puff control with 45 puffs of air, or E-cigarette-generated aerosol ± flavors. Each micrograph was photographed at 1000× total magnification (1920 × 1080 resolution) using a fluorescence microscope and converted to an 8-bit black and white image. (**B**) The percentage of the biofilm biomass (white area in A) was calculated using ImageJ. Each bar represents the mean ± SEM (*n* = 4). The red line represents the average biofilm percentage of the 0-puff control. RBG refers to red, blue, and green color images and B&W refers to black and white images.

Similarly, fluorescence microscopy was used to further investigate the effects of air and E-cigarette-generated aerosol ± flavors by characterizing the biofilm architecture of multispecies biofilms after 45 puffs of aerosol exposure. Figure 8A depicts representative images of multispecies streptococcal biofilm using the DNA stain SYTO 59 Red after exposure to 0 puffs (control) or 45 puffs of air or E-cigarette-generated aerosol ± flavors. Images are shown in color and in black and white. Figure 8B shows the quantification of the percent biofilm biomass (white area of the black and white images shown in Figure 8A). ImageJ analysis of the black and white images revealed no significant differences in the percent area of micrographs between the treatment groups of the multispecies biofilms.

### 3.5. Live–Dead Stain after Aerosol Exposure

**Figure 9:**
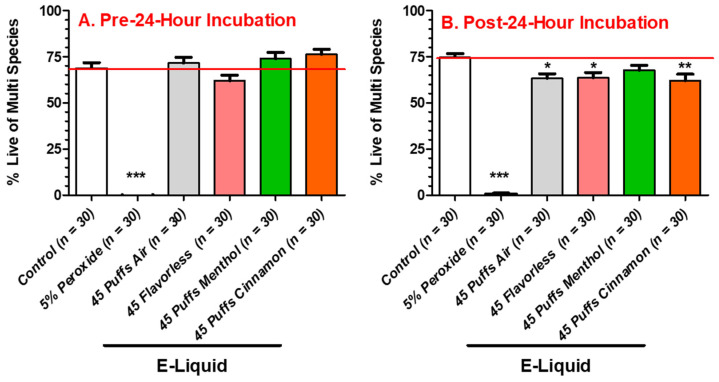
Aerosol-treated live/dead stain on multispecies biofilms. Percent viability of multispecies streptococcal biofilms exposed to 45 puffs of air or E-cigarette-generated aerosol ± flavors (menthol or cinnamon) before (**A**) and after (**B**) 24 h incubation. Each bar is the mean ± SEM (3 replicate experiments, *n* = 10 per replicate, total *n* = 30). Statistical significance was determined by one-way ANOVA with Bonferroni post hoc analysis where * = *p* < 0.05, ** = *p* < 0.01, and *** = *p* < 0.001 (comparing exposures of air to peroxide and E-cigarette-generated aerosols ± flavors). The red line represents the average baseline absorbance reading for the 0-puff control.

In order to measure the effects of air and E-cigarette-generated aerosols ± flavors on the viability of oral bacteria in multispecies biofilms, live–dead staining was conducted on biofilms treated under two conditions: aerosol treatment followed by a 24 h incubation (Pre), or a 24 h incubation followed by aerosol treatment (Post). Figure 9 shows the effects of 45 puffs of air and E-cigarette-generated aerosol ± flavors on the viability of multispecies biofilms as indexed by live/dead staining fluorescence. The percentages of live bacteria in the multispecies community after exposure to 45 puffs of air and the E-cigarette-generated aerosols ± flavors, but before 24 h incubation (Figure 9A), are similar to those of the 0-puff controls. This indicates that the streptococcal bacteria exposed to air and aerosols ± flavors recover during a 24 h incubation period. In contrast, the percentage of live bacteria in the multispecies community after 24 h incubation, followed by exposure to air and the E-cigarette-generated aerosols ± flavors (Figure 9B), reveals that 45 puffs of air or flavorless and cinnamon-flavored aerosols reduced bacterial viability, indicating that air and cinnamon-containing aerosols have a killing effect on the bacteria with no time to regenerate the biofilms. The results indicate that E-cigarette-generated aerosols (including air) have a slight effect on the viability of oral commensal biofilms, while having no effect on overall biomass (Figure 6A,B). Peroxide, as a positive control for killing, significantly inhibited multispecies viability, both before and after 24 h incubation.

## 4. Discussion

Oral commensal biofilms are essential for maintaining homeostasis of the oral cavity. Considering that the oral cavity is the first site of exposure for vaping, researching the effect of E-liquids is important for determining the potential hazards of E-cigarettes. Findings of past studies from our group have demonstrated the negative effect of flavored E-liquids on the planktonic growth and biofilm formation of oral commensal streptococci [36,37]. With this context, our study aimed to investigate the effect of flavored E-liquids on the viability (Figure 2) and hydrophobicity (Figure 3) of oral commensals; furthermore, we investigated the effect of flavored E-cigarette-generated aerosols on the biomass (Figure 5, Figure 6, Figure 7 and Figure 8) and bacterial viability in oral commensal biofilms (Figure 9). Our results indicate that exposure of oral commensals to cinnamon- and menthol-flavored E-liquids results in a bactericidal effect and increase in hydrophobicity. In contrast, exposure to cinnamon- and menthol-flavored E-liquids in their aerosolized forms does not dramatically alter the biomass or viability of individual or multispecies commensal biofilms.

The results of the live/dead stain after treatments with E-liquids (Figure 2) support the hypothesis proposed by Xu et al. (2022) [37] that direct exposure to flavored E-liquids has a bactericidal effect on the oral commensal streptococci tested. Previous research suggests that it is typical to observe some dead bacteria in a healthy biofilm [45], so we can corroborate that a healthy biofilm was established for the control condition (Figure 1). In addition, several other studies that investigated viability of biofilms with oral streptococci observed a ratio of live to dead cells in untreated biofilms between 4:1 and 2:1 [46,47,48]; the variation here may be attributed to differences in experimental conditions (i.e., media, time, species, etc.). The ratio observed in those studies agrees with the ratio of approximately 3:1 seen in our study and helps confirm that healthy biofilms were established, allowing us to draw more accurate conclusions on the impact that flavored E-liquids have on these multispecies commensal biofilms. With flavorless E-liquid exposure resulting in little to no change to the viability of the cells, we can conclude that the E-liquid humectants, PG and VG, are not the likely culprits that increase cell death within the established biofilms. The significant difference in cell death between the flavorless and flavored E-liquids suggests that there are specific compounds and properties within the flavoring agents increasing cell death. As previously observed by Xu et al. (2022), centrifugation following treatment of the streptococci with cinnamon flavor yields pellets of bacterial cells trapped with an amber-colored hydrophobic material [37]. Regarding the antimicrobial effect of cinnamaldehyde, one study found that exposure of *Escherichia coli* and *Staphylococcus aureus* to cinnamaldehyde results in damage to the bacterial cell morphology, membrane integrity, and permeability [49]. Other studies have found that exposure of *Streptococcus mutans* to cinnamaldehyde results in a decrease in biofilm formation, virulence gene expression, acid production, and aggregation while increasing hydrophobicity [50,51]. Based on those reports, it is possible that cinnamaldehyde, a component of the cinnamon flavor, contributes to cell death and decreased biofilm formation of the four oral streptococci tested. Regarding menthol, several studies have observed that menthol extract or essential oil had a bactericidal effect on *S. mutans* [52,53]. In addition, menthol is also known to be a hydrophobic solvent and is not particularly soluble in water [54], potentially contributing to this bactericidal effect. Further investigation involving biochemical analysis of cinnamon and menthol will yield more insight into the mechanism behind the bactericidal effect of these flavored E-liquids on oral commensal streptococci.

The oral microbiome typically exists in the form of a biofilm, which plays an important role in maintaining homeostasis in the oral cavity [24]. Cell surface hydrophobicity and hydrophobic interactions, among other physical and chemical properties, play an important role in the formation of these biofilms and mediating bacterial cell adhesion [55,56,57]. Specifically, hydrophobic interactions facilitate cell–cell interactions [56] and the adherence of cells to saliva-coated surfaces [57]. Furthermore, hydrophobicity has been observed to be a critical factor in biofilm formation, with a decrease in hydrophobicity correlating to a decrease in biofilm formation and vice versa [56,58]. Considering the importance of these interactions, a change in hydrophobicity could have serious implications; this may contribute to and help explain the negative effect of E-liquid flavors on oral commensal streptococci previously observed [36,37]. The results of this study demonstrate that all four oral streptococci tested are highly hydrophobic, with *S. oralis* being the most hydrophobic and *S. intermedius* being the least. Overall, most studies support the high levels of hydrophobicity for these four species [59,60,61,62,63], although one report observed *S. intermedius* to be less hydrophobic than other oral streptococci [64] and another study identified *S. mitis* as hydrophilic [61]. These discrepancies may be attributed to differences in experimental factors such as growth medium, organic solvent, and other unforeseen conditions. In addition, the lipodomes associated with these commensal streptococci include fatty acyl lipids, glycerolipids, and others [65]. These lipids most likely contributed to the baseline hydrophobicity observed in this study. It is also possible that these lipids could be interacting with menthol and cinnamaldehyde—which are also hydrophobic—potentiating the hydrophobicity. The results of the hydrophobicity assay in this study demonstrated that exposure to cinnamon and menthol flavors significantly increased the hydrophobicity of the four streptococci tested, while the base components PG and VG had a relatively small effect. Furthermore, cinnamon and menthol E-liquids cause a slight increase in hydrophobicity, although not significant when compared to the stock flavors (Figure 3), which is a reasonable outcome as the flavors are diluted to 25% in the E-liquid. Although these results differ from a previous study testing the effect of E-cigarette aerosols on oral commensals *Streptococcus sanguinis* and *S. gordonii* [66], which found that E-cigarette aerosols have no significant effect on hydrophobicity, this difference may be attributed to a difference in methodology: instead of exposing the bacteria directly to the aerosols, they pre-treated the growth medium. Overall, the results from this study regarding how E-liquids affect hydrophobicity may help explain the observations from the studies by Fischman et al. (2020) and Xu et al. (2022) that flavored E-liquids have a negative impact on oral commensal streptococci and their biofilm formation, while flavorless E-liquids—composed of PG and VG—do not [36,37]. Although previous research suggests that an increase in hydrophobicity should result in an increase in biofilm formation [36,37], these results coupled with the results of the live/dead staining experiments (Figure 2) demonstrate that the flavoring agents are not only changing the hydrophobicity of the bacteria but are also killing them; this explains the reduction in biomass observed in these previous studies. Nevertheless, it remains unclear how modest changes in hydrophobicity may alter biofilm formation and other processes [5].

While it is clear that exposure of the oral commensals to cinnamon- and menthol-flavored E-liquids results in a bactericidal effect and increases in hydrophobicity (Figure 2 and Figure 3), exposure to cinnamon- and menthol-flavored aerosols of E-liquids does not alter the biomass or viability of individual or multispecies commensal biofilms as dramatically. Crystal violet absorbance results of the individual streptococci exposed to E-cigarette-generated aerosols (Figure 5) reveal decreases in biofilm formation after 15, 30, and 45 puffs, especially for *S. intermedius*. This is supported by another study from our group [32] indicating that 100 puffs of E-cigarette-generated aerosol ± flavors bubbled directly into the BHI growth media inhibited planktonic growth during the log phase of individual streptococcal bacteria, especially *S. intermedius*. However, when the four bacteria were incubated as a multispecies community, E-cigarette-generated aerosols ± flavors had no effect on biofilm formation regardless of whether exposure occurred before or after 24 h incubation (Figure 6). This may be due to synergistic interactions within multispecies biofilms, not seen in single-species biofilms, which ultimately increase resistance, virulence, and overall biofilm survival [67]. In other words, multispecies biofilms are better able to overcome environmental stresses, such as E-liquid flavoring agents, compared to single-species biofilms. Visual inspection and quantification of biofilms/biomasses from both individual (Figure 7) and multispecies (Figure 8) streptococcal bacteria exposed to 45 puffs of E-cigarette-generated aerosols ± flavors failed to show any differences as compared to 0-puff controls. This is most likely due to the small sampling size and variations in the biofilms within and between coverslips.

The viability of multispecies biofilms (Figure 9) exposed to 45 puffs of E-cigarette-generated aerosols ± flavors fails to show any differences as compared to 0-puff controls when exposure occurs before 24 h incubation. In contrast, when exposure occurs after 24 h incubation, the viabilities of multispecies biofilms exposed to 45 puffs of air, flavorless E-liquid aerosols, and cinnamon-flavored E-liquid aerosols are mildly decreased as compared to 0-puff controls. Taken together, these results indicate that if exposure occurs before 24 h incubation, the multispecies biofilms have time to regenerate to control levels. However, if the multispecies bacteria are exposed after 24 h incubation, 45 puffs of air and E-cigarette-generated aerosols ± flavors have an immediate bactericidal effect. Interestingly, 45 puffs of air also decrease the viability of the multispecies community, but perhaps it is not air per se, but rather air movement (i.e., ventilation) that decreases bacterial viability. Qiu et al. (2022) [68] reported that increased ventilation inhibits the growth of bacteria on pork. In a test chamber (197″ L × 165″ W × 137″ H) with a ventilation rate of 0.56 m/s (at the chamber inlet) at 26 °C for a period of 24 h, non-specific bacterial growth on pork was significantly reduced by 10-fold. In comparison, our exposure chamber was much smaller (12″ L × 6″ W × 6″ H) with a ventilation rate of 0.09 m/s (at the chamber in-let; calculated from flow rate = 1.1 mL/minute) at 25 °C for 22.5 min (two 3 s puffs/minute; 45 puffs total), amounting to about a 90% reduction in chamber volume and 84% reduction in ventilation. Consequently, increased ventilation directed at the bacterial biofilms or the bacteria on the pork [68] is nearly identical. In our system, increased ventilation most likely caused significant evaporation of the thin biofilm surface moisture, resulting in fewer nutrients required for bacterial growth and possibly survival. Given the results (Figure 9), it remains unclear if the reduced viability noted following 24 h incubation is an artifact induced by increased ventilation during puffing. Whatever the reason for aerosol-induced reduction in biofilm viability post 24 h incubation, it is evident that a reduction in biofilm viability induced by exposure to aerosol is not as dramatic as direct biofilm exposure to E-liquids ± flavors (Figure 2). In a nicotine recovery study conducted in our laboratory (Supplementary File S1 and S2 [34]), utilizing the same E-cigarette, apparatus, and conditions used in the current study, it was determined that 4 µL of E-liquid is aerosolized per puff. After 25, 50, and 100 puffs, the percent recoveries of nicotine in the E-liquid were 0.19, 0.38, and 0.56%, respectively. Assuming that all components of the E-liquid are uniformly aerosolized, this equates to the same percentages/puff of flavoring agents in the BHI. In other words, biofilms are exposed to 0.34% flavoring agent after 45 puffs of a stock E-liquid containing 25% flavor. In contrast, biofilms bathed in BHI with 5% E-liquid containing 25% flavoring agent were exposed to 1.25% flavoring agent (Figure 2). Consequently, the direct effect of E-liquid represents an >3.6-fold increase in flavor concentration as compared to aerosol exposure (Figure 9), which would account for the more dramatic reduction in biofilm viability when exposed directly to E-liquid. These results are supported by the Xu et al. study [37] that reports dose-dependent decreases in biofilm formation for all streptococcal species exposed to either 0.25, 0.75, or 1.25% menthol or cinnamon flavoring agents.

Based on the results of this study, cinnamon- and menthol-flavored E-liquids have a bactericidal effect on the four oral streptococci tested (as a multispecies community) and significantly increase their hydrophobicity when the bacteria are exposed to them as liquids. To a lesser degree, the aerosols of cinnamon-flavored and flavorless E-liquids are also shown to reduce the viability of oral commensal biofilms while leaving the total biomass relatively unchanged. However, the experiments of this study have several limitations. First, this study was limited to four oral streptococci species and was unable to test the myriad oral bacterial species. These species were chosen because (i) they are primary colonizers of the oral cavity, (ii) they play an important role in the development of oral biofilms (dental plaque) [69], and (iii) to add to the current knowledge of past studies [36,37]. Second, although saliva would be the preferred growth medium, BHI medium was used because it supports the growth of oral commensal streptococci and is commonly used for in vitro assays [36,37,70]. Additionally, only two flavors were tested in this study, since those had previously been shown to have the most significant inhibitory effects on biofilm formation [37]; consequentially, these results may not be applicable across other E-liquid flavors. In regard to the experiments with aerosolized E-liquid, a maximum of 45 puffs was used. This may be less than the number of puffs most E-cigarette users take; one study found that the average number of puffs per day was 365 among E-cigarette users [71]. The reason 45 puffs was used in this study was for consistency with past studies from our group [34,35,36]. Furthermore, these studies did not consider the effect of the presence of pathogenic or opportunistic bacteria, which would more effectively mimic the oral microbial environment. Moreover, this study was limited to the effect of E-liquids or their aerosols, without taking into account the host presence.

Experiments are currently being designed to test the effects of E-liquids on polymicrobial biofilms with both commensal oral streptococci tested in this study and pathogens, such as *Porphyromonas gingivalis*, *Aggregatibacter actinomycetemcomitans*, *and Fusobacterium nucleatum*. With the addition of these pathogens, it will be possible to study the effect of E-liquids on the interspecies interactions of oral commensals and pathogens, in addition to changes in species composition and relative abundance. Furthermore, exposure to E-liquids ± flavors could lead to dysbiosis, the possibility of which could be explored in experiments involving both commensals and pathogens. Experiments are also currently underway to use techniques such as gas chromatography–mass spectroscopy to determine the compounds that are most frequently present in the flavoring agents for E-liquids; this will help determine which compounds are contributing to the toxicity and decreased growth patterns of oral microbes that have been observed.

## 5. Conclusions

In conclusion, this study demonstrates that menthol- and cinnamon-flavored E-liquids have a bactericidal effect on multispecies oral commensal biofilms and increase the hydrophobicity of oral commensal streptococci. In terms of hydrophobicity and viability, the results indicate that the cinnamon flavor has a more detrimental effect compared to the menthol flavor. They also demonstrate that flavorless and cinnamon-flavored aerosols have a bactericidal effect on oral commensal biofilms while having no effect on overall biomass. These findings extend previous observations from the study by Xu et al. that flavored E-liquids have a significant inhibitory effect on the growth and formation of single- and multispecies oral commensal biofilms [37]. Oral commensals are important for maintaining homeostasis in the oral cavity, and disturbances may lead to variations in the oral microbiome; this may prompt dysbiosis and increase susceptibility to oral disease [33]. These effects may also extend beyond the health of the oral cavity, considering the intertwined nature of oral and systemic health. Understanding the mechanism behind the impacts of flavored E-liquids on oral commensal biofilms in vitro provides an idea of how vaping may affect oral bacteria in vivo and the consequential effects on oral and systemic health.

## Figures and Tables

**Table 1:** Linear regression statistics for streptococcal bacteria exposed to 0, 15, 30, and 45 puffs of air or E-cigarette-generated aerosols ± flavors.

	Slope Value	*p*-Value *	R^2^
*S. gordonii*			
Air	0.03777 ± 0.1523	-	0.001336
Flavorless	−0.1644 ± 0.1569	NS	0.02379
Menthol	0.1688 ± 0.1438	NS	0.03106
Cinnamon	−0.7238 ± 0.2758	*p* < 0.01	0.1381
*S. intermedius*			
Air	0.4536 ± 0.05000	-	0.9763
Flavorless	−0.9701 ± 0.3070	*p* < 0.01	0.8331
Menthol	−0.6302 ± 0.2753	*p* < 0.01	0.7239
Cinnamon	−0.6302 ± 0.2753	*p* < 0.01	0.6158
*S. mitis*			
Air	1.150 ± 0.3194	-	0.2198
Flavorless	0.3234 ± 0.1490	*p* < 0.01	0.09665
Menthol	0.2252 ± 0.2133	*p* < 0.01	0.02527
Cinnamon	−0.02657 ± 0.2913	*p* < 0.01	0.0001808
*S. oralis*			
Air	0.6079 ± 0.1536	-	0.2583
Flavorless	−0.08662 ± 0.2611	*p* < 0.01	0.002554
Menthol	−0.1619 ± 0.1222	*p* < 0.01	0.03837
Cinnamon	0.4826 ± 0.1911	NS	0.1292

* significance from slope of air regression line. NS = no significance.

## Data Availability

All data are included in the manuscript.

## Supplementary Materials

The following supporting information can be downloaded at https://www.mdpi.com/article/10.3390/dj12080232/s1, File S1: Nicotine recovery study; File S2: Nicotine recovery study data. File S1 is a Word document which includes the Nicotine Calibration Curve along with Standard Curve Statistics and representative Chromatograms of Standards and Samples. File S1 also includes Aerosolization Curve, Nicotine Recovery and Pump/Chamber Parameters. File S2 is an Excel file which contains the Nicotine Recovery Data, Nicotine Standard Curve Statistics data, and Aerosolization data.
