# Supplementary material for: Effects of E-Liquids and Their Aerosols on Biofilm Formation and Growth of Oral Commensal Streptococcal Communities: Effect of Cinnamon and Menthol Flavors
Source: Dent J (Basel). 2024 Jul 23;12(8):232. doi: 10.3390/dj12080232 (PMC11353068; doi:10.3390/dj12080232)
Supplement: Supplementary file 1 [file dentistry-12-00232-s001.zip › File S1.pdf]

Supplemental Material

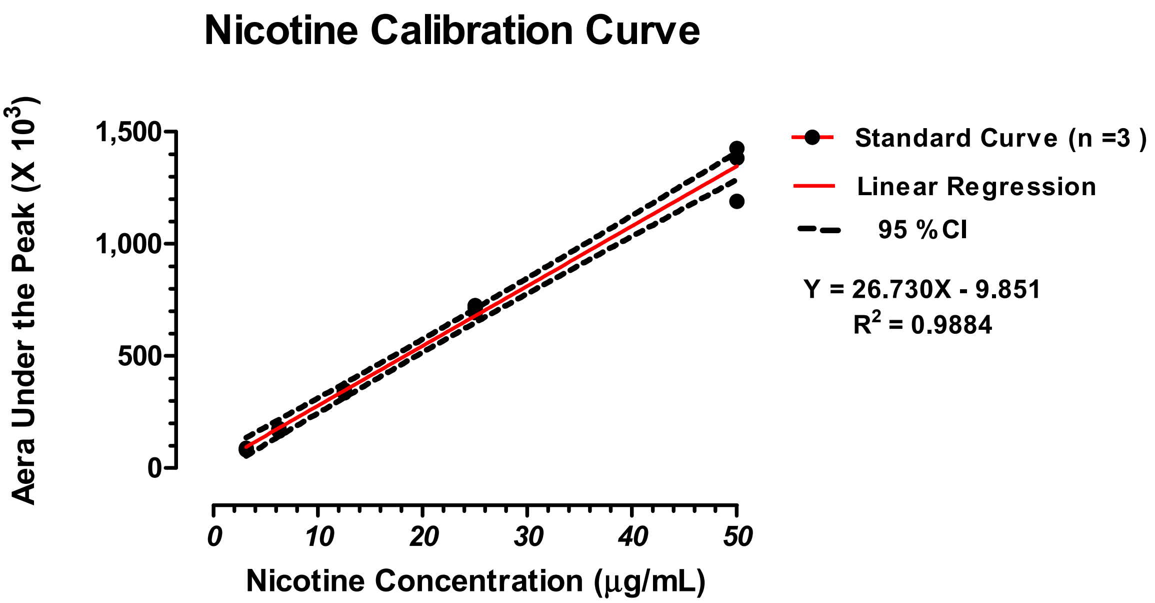

Nicotine Calibration Curve: Each point represents the mean (n = 3 of three replicate experiments; total n = 9) of area under the curve at nicotine concentrations of 3.125, 6.25, 12.5, 25 and 50 µg/mL. CI = confidence interval.

Standard Curve Statistics						
**Compound**	**Injected Concentration (µg/mL)**	**Retention Time(minutes) ***	**Measured Concentration(µg/mL) ***	**% Deviation from Injected Concentration**	**AreaUnder Peak***	**± 95 % Confidence Interval**
	3.13	9.85 ± 0.01	2.82 ± 0.08	9.788	85.22 ± 2.22	± 40.88
	6.25	9.76 ± 0.01	6.02 ± 0.13	3.664	170.81 ± 3.40	± 37.68
Nicotine	12.50	9.63 ± 0.01	12.35 ± 0.14	1.188	340.05 ± 3.66	± 32.22
	25.00	9.48 ± 0.00	26.20 ± 0.34	-4.788	710.19 ± 9.04	± 31.25
	50.00	9.30 ± 0.10	49.49 ± 2.73	1.027	1332.80 ± 72.95	± 60.84
Nicotine Straight Line Equation is Y = 26.730X – 0.9884; LOD (µg/mL) = 0.00003; LOQ (µg/mL) = 0.00009; R^2 =0.99; % RSD = 4.76						

*** = mean ± SEM where n=3; Limit of detection (LOD) and limit of quantitation (LOQ) were determined based on the calibration curve. %RSD = relative standard deviation (i.e., Coefficient of Variation) of the regression line.**

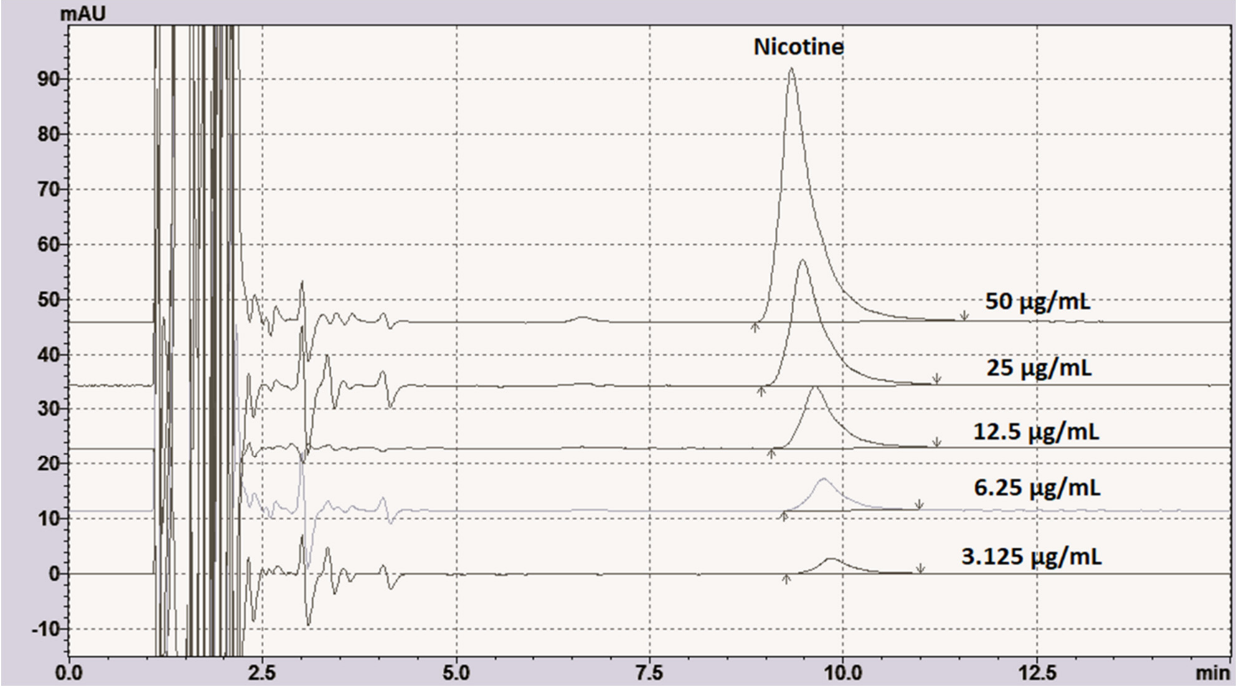

Representative Nicotine Chromatographs: Representative nicotine chromatographs were obtained using an HPLC coupled with photodiode array detection as previously described. A Shimadzu HPLC system (Columbia, MD) was used to quantitate nicotine and included the following: a photodiode array detector (SPD-M20A), dual pumps (LC-20AT), a column oven (CTO-20A), an in-line membrane degasser (DGU-20A3R) and a Rheodyne 7725I manual injector with a 20 µL loop (40 µL injection volume). Nicotine was separated on a Phenomenex (Torrance, CA, USA) 15-cm, Kinetex® 5 µm reversed phase C-18 column. Column temperature was maintained at 35 C. Nicotine was detected at ultraviolet (UV) wavelengths between 230 and 300 nm and quantifications were carried out at 260 nm. The mobile phase was delivered at a rate of 1 mL/minute in gradient fashion where mobile phase A consisted of 10% acetonitrile in 20 mM ammonium formate adjusted to pH 8.5 with 50% ammonium hydroxide and mobile phase B consisted of 100% acetonitrile. Mobile phase A decreased from 100% to 80% from 0 to 10 min, decreased from 80% to 20% from 10 to 20 min, increased from 20% to 100% from 20 to 21 min and remained at 100% till the end of the run time at 30 min. Mobile phase B increased from 0% to 20% from 0 to 10 min, increased from 20% to 80% from 10 to 20 min, decreased from 80% to 0% from 20 to 21 min and remained at 0% till the end of the run time at 30 min. **Cuadra, G.A.; Smith, M.T.; Nelson, J.M.; Loh, E.K.; Palazzolo, D.L. International Journal of Environmental Research and Public Health 2019, 16, 1669, doi:10.3390/ijerph16101669** [Reference 34 in the main paper].

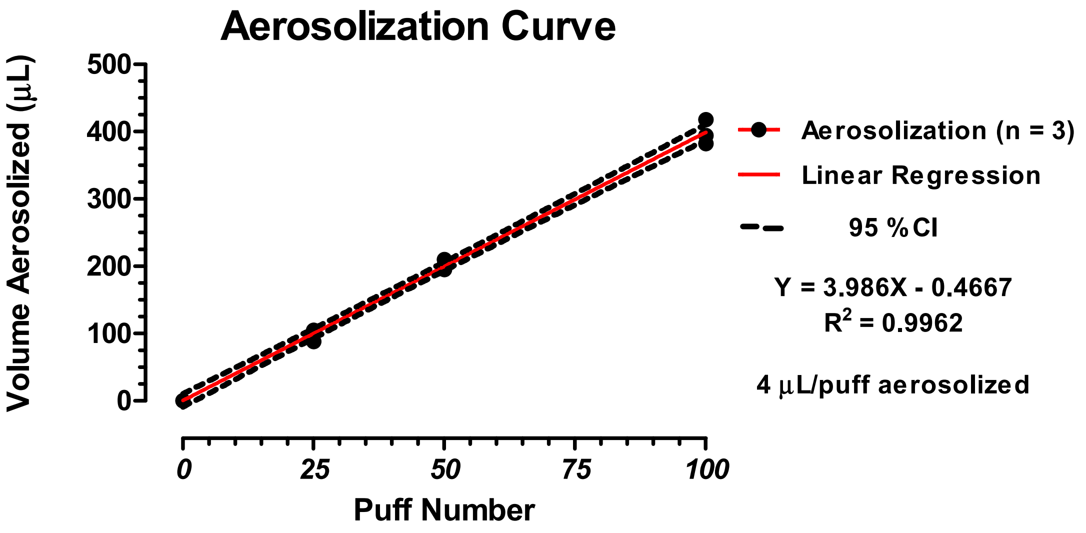

Aerosolization Curve: Each point represents the mean (n = 3 of three replicate experiments; total n = 9) of nicotine aerosolized after 25, 50 and 100 puffs. CI = confidence interval.

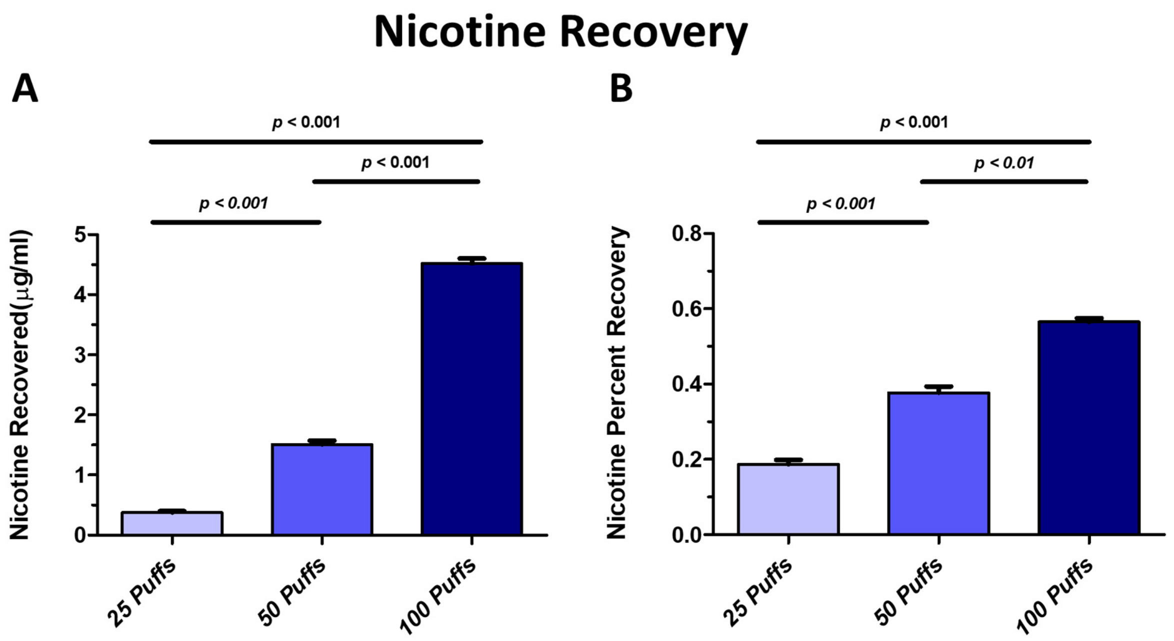

Nicotine Recovery: Each bar represents mean ± SEM (n = 3 of three replicate experiments; total n = 9) of absolute nicotine (μg/mL) recovered in BHI (A) and as a percent of nicotine in the chamber (B) after 25, 50 and 100 puffs.

**Pump/Chamber parameters and nicotine concentrations within the exposure chamber.**	
**Pump Flow Rate (mL/min)**	**1100**
**Puff Duration (s)**	**3**
**Puff Volume (mL)**	**55**
**Nicotine Concentration of E-Liquid (μg/mL)**	**20,000**
**Volume of E-liquid aerosolized/puff (μL)[1]**	**4**
**Nicotine (μg/puff)[2]**	**80**
**Nicotine Flow (μg/puff/mL) into Chamber[3]**	**1.45**
**Exposure Chamber Volume (cm^3)**	**5400**
**Nicotine Delivered to Chamber (μg)[4]**	
**25 puffs**	**2000**
**50 puffs**	**4000**
**100 puffs**	**8000**
**Nicotine Content (μg) in BHI (Mean ± SEM)[5]**	
**25 puffs**	**3.74 ± 0.02**
**50 puffs**	**15.07 ± 0.07**
**100 puffs**	**45.19 ± 0.08**
**Percent Recovery (Mean % ± SEM)[6]**	
**25 puffs**	**0.19 ± 0.01**
**50 puffs**	**0.38 ± 0.02**
**100 puffs**	**0.56 ± 0.01**

[1] Volume of E-liquid/puff aerosolized is calculated from the weight difference of the E-liquid before and after puffing, the density of E-liquid (1.18 g/mL) and the number of puffs (**Cuadra, G.A.; Smith, M.T.; Nelson, J.M.; Loh, E.K.; Palazzolo, D.L. International Journal of Environmental Research and Public Health 2019, 16, 1669, doi:10.3390/ijerph16101669**) [Reference 34 in the main paper]. [2] Nicotine (μg/puff) represents the fraction of the original E-liquid concentration (20,000 μg/mL) in one puff (4 μL). [3] Nicotine flow is derived from the amount of nicotine pumped into the chamber during each 3 s puff (55 mL). [4] These values assume that all nicotine remains within the chamber, which is not the case since the exposure chambers are equipped with an aerosol outlet directly opposite the aerosol inlet, to allow venting of the exposure chambers. Furthermore, additional aerosol escapes the chamber immediately after 25 and 50 puffs when the exposure is briefly opened to remove the 25- and 50-puff plates from the chamber, respectively. [5] Mean nicotine content (n =3 of three replicate experiments) in 10 mL of BHI inside the chamber as determined by HPLC-PAD. [6] Mean % recovery (Nicotine content in BHI/Nicotine content delivered to Chamber).
